# Acute Sclerokeratitis After Cataract Surgery: Treatment with Topical Use of Cyclosporine – A

**DOI:** 10.2174/1874364100802010031

**Published:** 2008-03-08

**Authors:** K Doulas, C Pantazopoulou, D Feretis

**Affiliations:** Department of Ophthalmology, General Hospital of Patras, Patras, Greece

## Abstract

We would like to report an interesting case of acute sclerokeratitis following cataract surgery treated with topical use of Cyclosporine-A. A 61-year old woman with a past history of scleritis in her right eye had an uneventful phaco surgery in her right eye for cataract removal *via *a corneoscleral incision. Eight months after the initial surgery the patient had a sudden decrease in her visual acquity in the right eye with marked inflammatory signs in the sclera and cornea adjacent to the entry-site of the phaco surgery. Initially the patient was treated with systemic corticosteroids but due to serious side effects topical cyclosporine-A was added instead. Five months later the patient has a significant improvement in terms of visual acquity with marked reduction in the inflammatory signs both in sclera and corneal tissue. We think that topical Cyclosporine-A with its potent immunomodulating effects seems to be of benefit in those cases where systemic corticosteroids are contraindicated or have serious side-effects.

## INTRODUCTION

Surgically induced necrotizing sclerokeratitis is a rare destructive form of scleritis which concerns more and more frequently the ophthalmic community due to the rapid increase of patients having cataract surgery. Cyclosporine is a potent immunomodulator that inhibits T-cell activation through inhibition of synthesis of interleukin-2, a growth factor for T lymphocytes. We would like to report an interesting case of acute sclerokeratitis following phaco surgery treated with topical use of Cyclosporine-A.

## MATERIALS AND METHODS

**Fig. (1).**
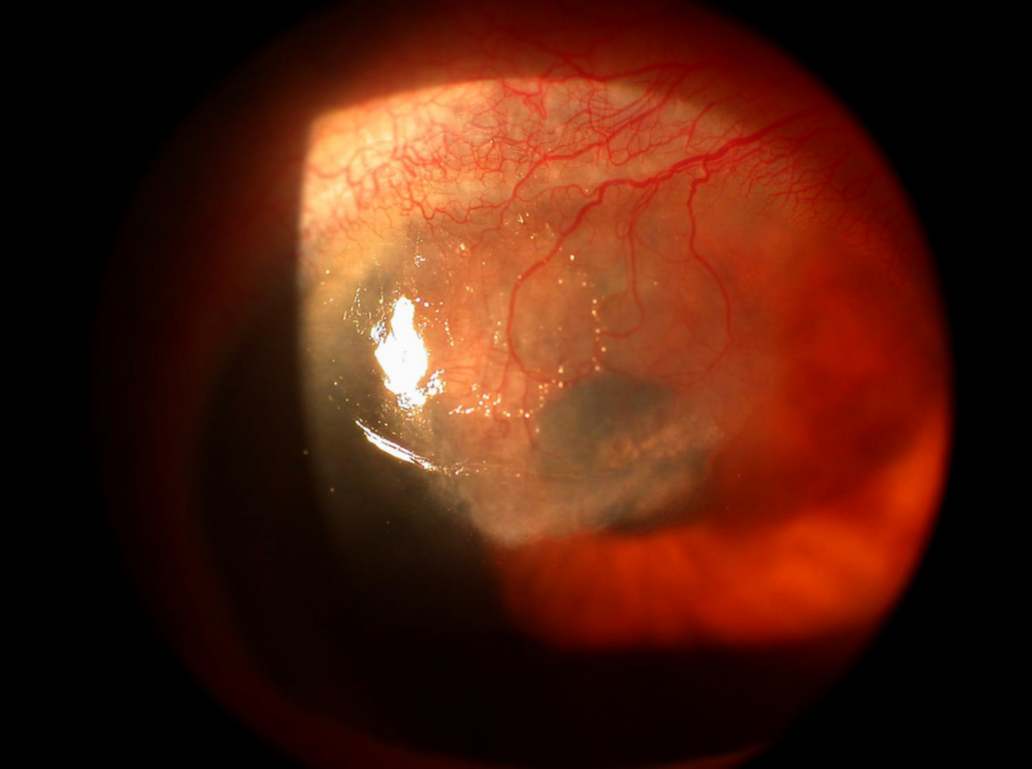
Colour photo of the patient right eye. Intense inflammation of scleral tissue as well as neovascularization of the cornea adjacent to the cataract incision site.

A 61 year-old female patient had phaco surgery for cataract removal in her right eye *via *a corneoscleral tunnel incision 3mm in length. The medical history of the patient was free of any serious disease. The ocular history included two episodes of anterior nodular scleritis in the right eye for which the work-up showed no specific cause and were both successfully treated with the use of systemic NSAIDS. The phaco surgery was uneventful and the postoperative medication included the use of dexamethazone eye drops every 2 hours and ofloxacin eye drops every 2 hours for ten days. The postoperative course of the patient was asymptomatic until 8 months after surgery when the patient presented in the outpatient department of the Eye Clinic with severe pain, redness, lacrimation and visual loss in her right eye. The best corrected visual acquity in the right eye was V=1/10. Slit-lamp examination inflammatory signs in the sclera with dilated episcleral and conjuctival vessels adjacent to the entry site of the phaco surgery. There were also inflammatory signs in the cornea adjacent to the entry site including growth of neovascular tissue between 9 and 11 clock creeping through corneal stroma towards the centre of the cornea and early corneal thinning at this area (Fig. **1**).

There were no cells or flare in the anterior chamber and fundus view by an indirect ophthalmoscopy was normal. The intraocular pressure by applanation tonometry in the right eye was 17mmHg. Scrapings from the lesion were taken and sent to the microbiological lab for stain and culture. No organism was detected. A complete systemic work-up by an internist and rheumatologist failed to reveal any systemic disorder. A laboratory work-up (haematology, serology and immunology profiles) was done and the results were either normal or negative. Based on clinical presentation and negative systemic findings acute sclerokeratitis of non necrotizing variety was diagnosed and the patient was started initially on oral prednisolone 75 mg/day and frequent use of artificial tears. During the steroid therapy the patient showed little improvement but she developed cushinoid features and she refused to continue. The corticosteroids were tapered gradually and topical suspension of cyclosporine-A 1% prepared by one of us (by admixing 10 ml of oral solution of Sundimnum Neoral 500mg/ml with 90ml of artificial tears [tears natural II –Alcon]) was added instead. The patient was given instructions to take the cyclosporine drops four times daily and evaluated every month. Until now the patient has been on cyclosporine drops for a period of five months.

## RESULTS

**Fig. (2).**
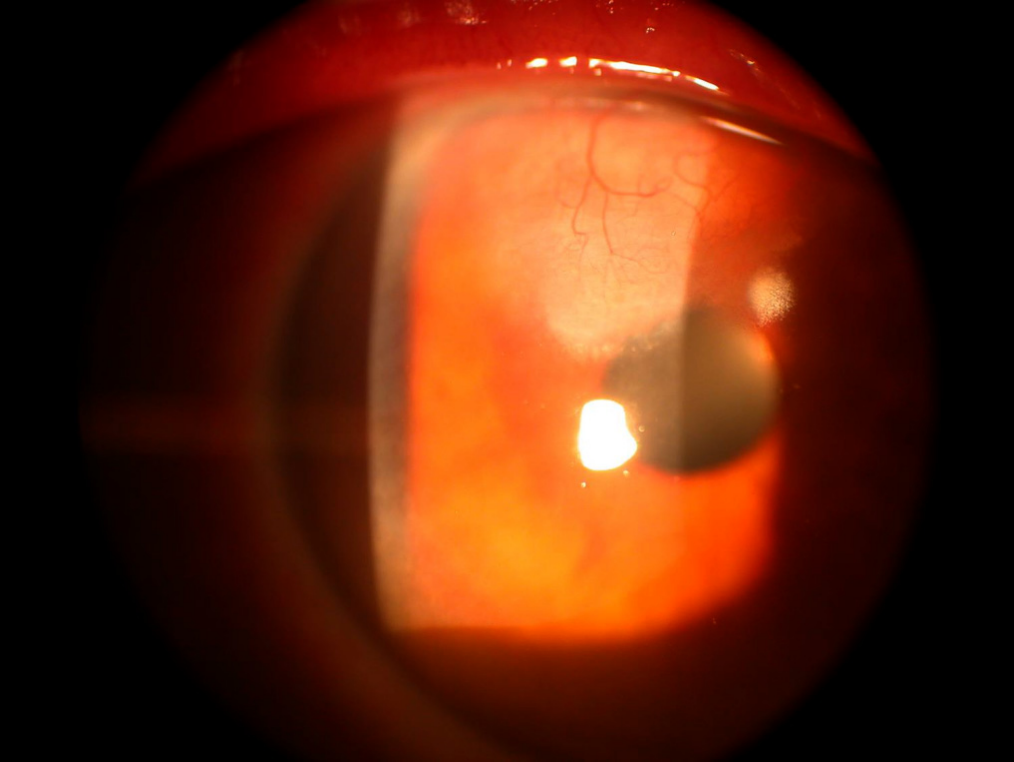
After treatment with topical Cyclosporine-A 1% there is reduction of the neovascularization in the cornea and the appearance of scar tissue in upper corneal stroma.

5 months after the beginning of treatment with topical Cyclosporine-A 1% the patient is in good condition and the the use of cyclosporine is tolerated very well. Slit-lamp examination of the patient right eye showed a marked reduction of inflammatory signs both in sclera and corneal tissue and a gradual disappearance of neovascular tissue in the cornea. The cornea shows a reduction in thickness adjacent to the initial cataract incision and a mild amount of scarring into he same area (Fig. **2**).

The visual acquity of the patients right eye shows a moderate improvement V=3/10 because of irregular astigmatism as a result of peripheral corneal thinning and scarring.

## DISCUSSION

Surgically induced necrotizing scleritis SINS, is a destructive inflammation of the sclera which can be complicated by keratitis, corneal ulcer, and ultimately perforation of the cornea. In about 96% of cases SINS is of necrotizing variety [1]. Usually the scleral inflammation is developed adjacent to a surgical wound. Many different types of ocular surgery like cataract extraction, glaucoma, strabismus, retinal detachment surgery, pterygium excision, can induce SINS [2]. O these cataract surgery (phaco or ECCE) through a limbal incision comprises the largest subgroup. The reported latent period between ocular surgery and the onset of SINS is about 9 months but it may also occur after several years in a small group of cases. In from 63-90% of patients SINS has been associated with systemic diseases like rheumatoid arthritis and other vasculities [3]. The pathophysiological mechanisms of SINS have not been established yet. Diaz-Valle *et al.* found that patients without an underlying autoimmune disease (as our patient) had the mildest form of scleral ulceration compared to those with a systemic disease who developed necrotizing scleral ulceration [4]. Neutrophile and macrophage infiltration around the vessel wall and the corneal stroma represents the first response to the surgical trauma. The co-existence of immune complex deposition in the cornea, episcleral and scleral vessels is evidence of vasculitic pathology. T-cell mediated immunological responses also play an important role in SINS of the necrotizing variety. Histological evaluation of the severe cases showed the presence of T-helper cells, up-regulation of HLA-DR expression on the surface of scleral fibroblasts, and epithelial cells of the corneal ulcer and local immunoglobulin deposition (IgM, IgG). Addionally the fact that SINS is developed more frequently following multiple surgical events suggests that it may be the result of a hypersensitivity reaction directed against an antigen revealed or altered by tissue injury. The increased expression of TNF-a and MMP-9 (a proinflammatory cytokine and tissue degrading protease) in scleral tissue of patients with SINS supports this hypothesis [5]. Earlier studies showed a correlation between different suture materials and SINS but this correlation has not been verified by other reports. Primary vascular closure and ischemia play a role but are unlikely to be important initiating events because in most cases SINS does not occur in the immediate postoperative period. Also the rapid response to immunosuppressive drugs implies an involvement of the immune system. The deregulation of all these immune mechanisms may lead to the development of SINS. Finally we have to consider that local ocular factors such as infections and conjuctival ulcers can also cause SINS *via *proteolytic enzymes and toxins and need to be excluded during the evaluation of the disease [6]. Management of SINS includes a preoperative evaluation to rule out any systemic disease. Intraoperatively, although there is no general consensus, clear corneal incision may be preferable to corneoscleral since the disease is probably triggered by vascular inflammation. Post-operatively the use of high dose systemic corticosteroids is the first line of treatment. The use of NSAIDS is not effective. Cyclosporine-A is a potent immunomodulator that acts locally when administered at the ocular surface. Cyclosporine inhibits T-cell activation through its action as calcineurin inhibitor. This action results in the inhibition of the synthesis of interleukin-2, a growth factor for T lymphocytes Topical use of Cyclosporine-A is becoming popular for the prevention of allograft rejection in corneal transplants and for controlling immunologic or allergic disorders of the ocular surface [7]. Clinical studies have documented that cyclosporine after topical application accumulates at the ocular surface and cornea reaching concentrations that are sufficient for immunomodulation. It has also been used as an alternative treatment to topical steroids in the treatment of the inflammatory response in progressive corneal melting. In our case a topical suspension of Cyclosporine-A was used since the previous steroid treatment caused severe cushinoid features and the patient refused to continue on steroid therapy. Finally, in cases of progressive corneal and scleral melting we believe that despite treatment with topical Cyclosporine-A, a surgical management will probably be needed. In conclusion the Ophthalmologist should remain alert about the possible occurrence of SINS. The development of SINS after uneventful cataract surgery can be the first manifestation of a serious systemic disease, therefore prompt and early diagnosis and aggressive treatment should be initiated in order to maximize a successful ocular and medical outcome. Given the success of topical Cyclosporine-A in several ocular diseases caused by T-cell dysfunction it follows that topical use of Cyclosporine-A may have value in the treatment of SINS in the cases where systemic corticosteroids are contraindicated or have serious side-effects.
